# Empowerment and HIV Risk Behaviors in Couples: Modeling the Theory of Gender and Power in an African Context

**DOI:** 10.1089/whr.2019.0020

**Published:** 2020-04-21

**Authors:** Makhabele Nolana Woolfork, Ashley Fox, Andrea Swartzendruber, Stephen Rathbun, Joel Lee, Jane N. Mutanga, Amara E. Ezeamama

**Affiliations:** ^1^ Department of Epidemiology and Biostatistics, College of Public Health, University of Georgia, Athens, Georgia, USA.; ^2^ Department of Public Administration, University at Albany SUNY, Albany, New York, USA.; ^3^ Department of Health Policy and Management, College of Public Health, University of Georgia, Athens, Georgia, USA.; ^4^ Department of Psychiatry, Michigan State University, East Lansing, Michigan, USA.

**Keywords:** empowerment, HIV, sub-Saharan Africa, Theory of Gender and Power

## Abstract

***Background:*** Young women and girls in Eastern and Southern Africa are at elevated risk of acquiring human immunodeficiency virus (HIV) compared with men, largely due to power dynamics within heterosexual relationships that contribute to HIV risk behaviors. Few studies employ a comprehensive framework to examine divisions between men and women and HIV risk behaviors in an African context. Thus, we examined associations between levels of women's empowerment and HIV risk behaviors applying the Theory of Gender and Power.

***Methods:*** We used logistic regression (adjusted odds ratios or AORs) to assess associations between women's empowerment indicators and HIV risk behaviors (multiple sexual partners) and self-efficacy (ability to negotiate sex/sex refusal) with couples data (*n* = 12,670) from Malawi, Namibia, Zambia, and Zimbabwe.

***Results:*** Specifically, key drivers of high levels of empowerment among women were household decision-making involvement, female economic independence, and rejecting all reasons for wife-beating. Furthermore, higher levels of women's empowerment in coupled relationships was associated with safer sex negotiation in Malawi (AOR = 1.57, *p* < 0.05) and Zambia (AOR = 1.60, *p* < 0.0001) and sex refusal in Malawi (AOR = 1.62, *p* < 0.0001) and Zimbabwe (AOR = 1.29, *p* < 0.05). However, empowerment was not associated with the likelihood of the male partner having multiple sexual partners across all countries studied.

***Conclusions:*** These findings provide evidence that high levels of women's empowerment were associated with safer sex practices, although this varied by country. Policymakers should incorporate empowerment indicators to address women's empowerment and HIV prevention within African couples.

## Introduction

Of the estimated 20.6 million people living with human immunodeficiency virus (HIV) in Eastern and Southern Africa in 2018, the majority of them were female adults and adolescents.^1^ On average, young women between 15 and 24 years acquire HIV 5–7 years earlier than young men.^2,3^ Overall, high gender inequality correlates with countries having predominantly heterosexual epidemics.^4^ Women and girls in sub-Saharan Africa (SSA) are at elevated risk of acquiring HIV due to earlier age at sexual debut, transgenerational sex, gender-based violence (GBV), lower access to education than young men, and the absence of essential health services.^2,5–8^ At the individual and interpersonal levels, these factors drive power dynamics in heterosexual relationships and lead to HIV risk behaviors such as multiple sexual partners and low condom use.^9^

Solutions that address female disempowerment are expected to reduce HIV risk in women.^10^ Public health research and programs have addressed gender inequities with female-centered programs, although these have sometimes been criticized for emphasizing a perspective that views women as victims and men as perpetrators.^11,12^ Other researchers have addressed structural factors linked to gender imbalances with programs such as those in microfinance and education.^11,13^

Further, couples-based skills-building, couples' HIV testing and counseling, “Treatment as Prevention,” and a scale-up of male circumcision programs acknowledge men's vulnerability to HIV.^14^ As a multidimensional construct, empowerment involves structural divisions of power, labor, and broader social conditions that influence men and women.^15,16^ One theory that combines gender dynamics operating at different levels is the multidimensional Theory of Gender and Power (TGP), which is applicable to HIV prevention in women.^15,17^

Few studies have employed a comprehensive framework such as the TGP to examine divisions between men and women and HIV risk behaviors in couples in an African context. Moreover, policymakers in Eastern and Southern Africa rarely include women and girls in national strategic plans for gender equality and HIV/acquired immunodeficiency syndrome prevention.^12,18^

It is thus important to examine which women's empowerment indicators influence associations with sexual behaviors to inform HIV prevention efforts. Therefore, our research assessed the association between TGP constructs for empowerment in married/cohabitating women in coupled relationships and HIV risk behaviors. We hypothesized that women with higher levels of empowerment would experience lower likelihood of multiple sexual partners in the relationship and higher likelihood of self-efficacy (ability to ask a partner to wear a condom given a sexually transmitted infection [STI] and ability to refuse sex) compared with women with lower levels of empowerment.

## Materials and Methods

### Study design and population

This study was a cross-sectional analysis of couples data from the Demographic and Health Survey (DHS) with men and women aged 15–64 years in Malawi, Namibia, Zambia, and Zimbabwe. These countries were selected because (1) respondents had complete empowerment and HIV risk behavior information, (2) recent data were available (2010–2014), (3) the geographic location was in Southern or Eastern Africa, and (4) HIV prevalence was 10% or higher in the sample.

**FIG. 1.**
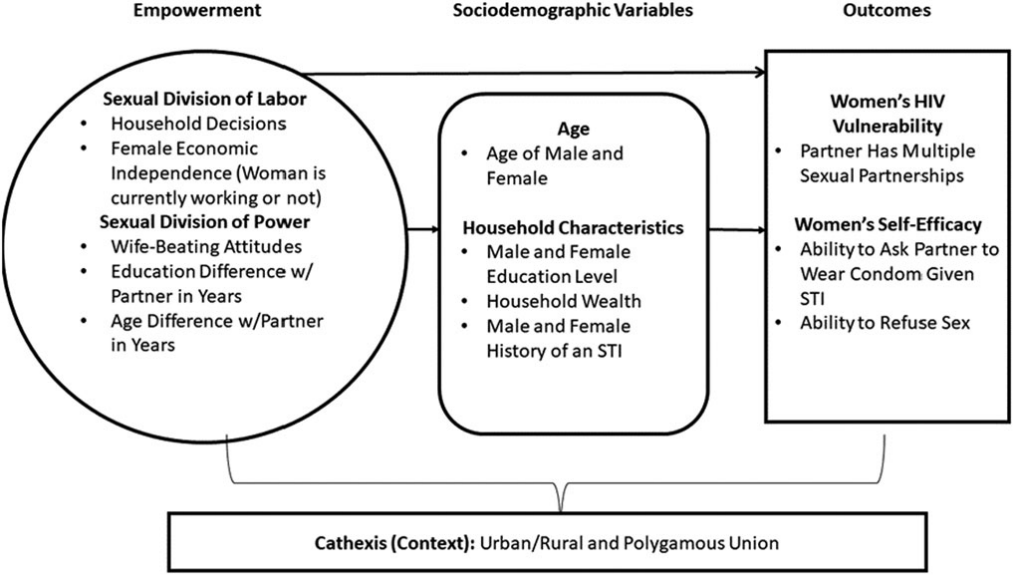
Conceptual framework using the Theory of Gender and Power; associations between women's empowerment, and HIV-related behaviors in African couples. HIV, human immunodeficiency virus.

The DHS is a cross-sectional nationally representative household survey implemented in low- to middle-income countries around the world,^19^ with a two-stage sample of households and individuals, mainly children, women (aged 15–49 years), and men (aged 15–64 years).^20,21^ The DHS randomly selects households at district or province levels, and individuals are picked at random within households for interviews and clinical tests.^20^ Individuals for whom the primary determinant, empowerment, could not be defined and those without information on the outcome measures were excluded (Fig. 1). We assigned higher values to categories of women with greater empowerment.^16^

Typically, DHS uses a two-stage sample of households and individuals designed to be representative of households at a national and subnational level.^20,21^ These samples are stratified by geographic region and by urban/rural areas within each region. Within each stratum, the sample design specifies an allocation of households to be selected. Most DHS surveys establish a set number of households per cluster, determining the number of clusters to be selected. In the first stage, primary sampling units are selected to form a survey cluster. During the second stage, a household listing is conducted for each cluster, and a set number of households is selected with probability proportionate to size.^20^

We applied sampling weights in all analyses for the following reasons: (1) to account for variations in selection probabilities for households across clusters, (2) to adjust for possible nonresponse rate across households within various clusters, and (3) to address analytically the possible oversampling of certain subgroups and thus derive nationally representative samples of the population of interest.

### Outcomes

#### Multiple sexual partnerships

In assessing HIV risk, we measured the number of multiple sexual partnerships, not including the spouse/partner, reported by the man in the past 12 months before the survey. We calculated frequencies for man's nonmarital multiple sexual partnerships and dichotomized responses as a “Yes” or “No” answer.

#### Ability to ask partner to wear a condom given an STI

This survey question asked women, “Can you/a woman ask a man to wear a condom if he has an STI?” We placed “Yes” answers in a separate category from “No” answers, which included “Don't Know” responses.

#### Ability to refuse sex

The next question asked women, “Can you/a woman refuse sex?” We placed “Yes” answers in a separate category from “No” answers, which included “Don't Know” responses.

### Predictor

#### Women's empowerment index

We defined women's empowerment as incorporating household decision-making, attitudes toward wife-beating, female economic dependence, and age and educational differences between partners using the TGP conceptual framework (Fig. 1), adapted from Wingood and DiClemente, and the Survey-based Women's Empowerment Index.^16,17^ We addressed our hypothesis by weighting each indicator equally, assigning responses for each survey response as high versus low levels of empowerment (“1” as high, “0” as low), and adding all numerical components to create a final composite empowerment score by country. Finally, we used the median value of each index by country to create a dichotomous variable with “high” (scores above the median) and “low” (median score and below) categories.^22^

We conceptualized the sexual division of labor as a woman's ability to make decisions about household purchases and their economic independence from her husband.^23^ We coded women who made decisions alone or jointly with their partner as having high levels of empowerment (“1”) and women whose husband/partner or someone else made decisions as having low levels of empowerment (“0”).^23^ We defined female economic independence as whether the woman reported that she had worked in the past 12 months (regardless of location or type of work) before the survey (coded as “1”) or not (coded as “0”).

We conceptualized the sexual division of power using the dimensions of attitudes toward violence against women and age and educational differences between men and women. Responses to a question asking whether wife-beating is ever justified, with several reasons offered, were coded as “1” for high levels of empowerment when respondents answered “No” and as “0” for low levels of empowerment to answers of “Yes” and “Don't Know.” We then created a variable comparing empowered and disempowered respondents.^23^ We calculated age difference by subtracting the female respondent's age from the partner's reported age and created categories to reflect age ranges between partners. We created a dichotomous variable comparing scenarios where partners are the same age, the woman is older, or the partner is up to 9 years older versus those where the man is 10 or more years older than the woman.^17,24–27^

We calculated the difference in years of education by subtracting the female's years of education from the male's years of education. Subsequently, we created a new variable with four categories^28^: (1) male partner with lower level of education than the female partner, (2) male and female partner with same level of education, (3) male partner with 1–3 years of education more than the female partner, and (4) male partner with 4 or more years of education more than the female partner. Finally, we compared scenarios where the man had fewer or the same number of years of education as the woman versus the woman had fewer years of education similar to previous research.^29,30^

#### Sociodemographic variables

As per our model (Fig. 1), we adjusted for specific variables associated with HIV risk behaviors or HIV acquisition, including age of the man and woman,^17,31^ educational level of both partners,^17,30,32^ household wealth,^29,33^ partners' history of an STI,^17,34^ place of residence,^35,36^ and polygamy (cathexis).^37,38^ Each variable had a corresponding reference group to depict high versus low levels of empowerment. We measured and categorized women's and men's ages in years according to the DHS: 15–24, 25–29, 30–34, 35–39, 40–44, 45+, and 50+ years. We separated education level for both genders into four categories: none (reference), some primary, completed primary/some secondary, and completed/more than secondary.

We used the DHS wealth index to measure household wealth in five categories, then collapsed categories into tertiles for simpler analysis: poor (reference), middle, and rich. Previous STI infection (“Yes”/“No”) was assessed by three questions: During the past 12 months, have you had a disease that you got through sexual contact? Did you have genital sores or ulcers in the past 12 months? Did you have genital discharge in the past 12 months? A person had an STI if he/she responded “Yes” (reference) to all three questions; otherwise, responses were categorized as “No.”

We assumed that women in urban dwellings might experience less harmful traditional norms, and thus, we compared urban dwellers versus rural dwellers. Finally, we separated polygamous unions into two categories: “Yes” (more than one wife) as the reference group and “No” (one wife) to test the assumption that women in polygamous unions may experience lower levels of empowerment than women who are not in polygamous relationships.

### Statistical analysis

Each statistical assessment was performed by country. We conducted an assessment for effect modification (association difference by level of a third variable) and mediation (association depends on the presence or absence of a third variable) in the association between levels of empowerment and HIV risk behaviors by wealth tertile *a priori*. The results did not yield any relevant findings (not shown).

First, we applied DHS sample weights to all analyses of couples data to account for the random sampling design and lower response rates for men.^21^ Then, we described each country with descriptive statistics and univariate analyses for mean age difference and used chi-square analysis to test differences in frequencies of other variables by the level of empowerment. Next, we assessed the relationship between individual empowerment indicators and the three outcomes of interest using unadjusted odds ratios (UORs).

As a result of our chi-square tests, we adjusted for the following country-specific variables in our final multivariable logistic regression models:

Malawi: woman's age, wealth index, place of residence, and polygamous union.

Namibia: woman's age, wealth index, place of residence, and man's history of an STI.

Zambia: man's age, wealth index, place of residence, woman's history of an STI, and polygamous union.

Zimbabwe: woman's age, wealth index, place of residence, and woman's history of an STI.

Finally, for multivariable analyses by country, we built a logistic regression model from which adjusted odds ratios (AORs) and 95% confidence intervals were calculated to quantify the association between indicators of women's empowerment and HIV risk behaviors. Also, our models generated *p*-values to indicate statistical significance (*p* < 0.05). We used SAS^®^ software, version 9.4, for all analyses.^39^

## Results

Table 1 presents weighted frequencies of couples by demographics of interest for each country (*n* = 12,670). Overall, self-reports of STIs were much higher for women than men. Zimbabwe had the highest proportion of women involved in decision-making solely or jointly (67%) but had the second-highest percentage (41%) reporting that one or more reasons justified wife-beating. In contrast, most (84%) women in Malawi were not involved in household decisions, but Malawi also had the largest share of women who rejected all reasons for wife-beating (88%). The mean age difference between partners was ∼5 years, with men being older, which is a risk factor for women's HIV risk. Overall, women's responses in all countries except Zambia (52%) were in the low-level empowerment categories. However, reported monogamy by a man was high in the past 12 months (86%–94%), most women said they/a woman can ask a partner to use a condom if he has an STI (83%–97%), and >70% of women said they/a woman can refuse sex.

**Table 1.** Sociodemographic Characteristics in Couples Aged 15–64^+^ Years in four Eastern and Southern African Countries

	Malawi 2010 (*n* = 2849)	Namibia 2013 (*n* = 865)	Zambia 2013–14 (*n* = 6039)	Zimbabwe 2010–11 (*n* = 2917)
	Frequency (weighted) and percentage			
Woman's age categories (years)				
15–24	996 (34.9)	133 (15.4)	1476 (24.4)	938 (32.1)
25–29	817 (28.7)	145 (16.7)	1388 (23.0)	714 (24.5)
30–34	433 (15.2)	146 (16.9)	1232 (20.4)	545 (18.7)
35–39	327 (11.5)	148 (17.1)	938 (15.5)	390 (13.4)
40–44	173 (6.1)	104 (12.0)	610 (10.1)	216 (7.4)
45+	103 (3.6)	189 (21.8)	395 (6.5)	114 (3.9)
Man's age categories (years)				
15–24	378 (13.3)	45 (5.2)	434 (7.2)	291 (10.0)
25–29	658 (23.1)	105 (12.1)	991 (16.4)	639 (21.9)
30–34	634 (22.2)	151 (17.5)	1240 (20.5)	615 (21.1)
35–39	499 (17.5)	140 (16.2)	1195 (19.8)	560 (19.2)
40–44	292 (10.3)	125 (14.4)	932 (15.4)	389 (13.3)
45–49	237 (8.3)	117 (13.6)	626 (10.4)	240 (8.2)
50+	151 (5.3)	182 (21.0)	621 (10.3)	183 (6.3)
Woman's education level				
Some primary	1965 (69.0)	154 (17.8)	2522 (41.8)	393 (13.5)
Completed primary/some secondary	692 (24.3)	429 (49.6)	2838 (47.0)	2395 (82.1)
Completed/more than secondary	192 (6.7)	282 (32.6)	679 (11.2)	129 (4.4)
Man's education level				
Some primary	1663 (58.4)	169 (19.5)	1512 (25.0)	286 (9.8)
Completed primary/some secondary	718 (25.2)	393 (45.4)	3195 (52.9)	2323 (79.6)
Completed/more than secondary	468 (16.4)	303 (35.1)	1332 (22.1)	308 (10.6)
Household wealth index				
Poor	896 (31.4)	288 (33.3)	1947 (32.2)	898 (30.8)
Middle	895 (31.4)	278 (32.1)	1790 (29.6)	1012 (34.7)
Rich	1058 (37.1)	299 (34.6)	2303 (38.1)	1007 (34.5)
Place of residence				
Urban	518 (18.2)	578 (66.9)	2551 (42.2)	998 (34.2)
Rural	2331 (81.8)	286 (33.1)	3488 (57.8)	1919 (65.8)
History of an STI (yes)				
STI past 12 months woman	336 (11.8)	111 (12.8)	253 (4.2)	284 (9.8)
STI past 12 months man	172 (6.0)	41 (4.8)	318 (5.3)	184 (6.3)
Polygamous union^a^				
Yes	208 (7.3)	19 (2.2)	507 (8.4)	171 (5.9)
No	2641 (92.7)	846 (97.8)	5532 (91.6)	2746 (94.1)
Woman's empowerment indicators				
Participation in decision-making				
Involved in all household decisions	449 (15.8)	510 (58.9)	2909 (48.2)	1963 (67.3)
Not involved in all household decisions	2400 (84.2)	355 (41.1)	3130 (51.8)	954 (32.7)
Female economic independence				
Currently working	1672 (58.7)	457 (52.8)	3350 (55.5)	1096 (37.6)
Not currently working	1177 (41.3)	408 (47.2)	2689 (44.5)	1821 (62.4)
Attitudes toward wife-beating				
None of five reasons are justified	2502 (87.8)	628 (72.6)	3141 (52.0)	1706 (58.5)
One or more reasons are justified	347 (12.2)	237 (27.4)	2898 (48.0)	1211 (41.5)
Age difference with partner in years^b^				
Partner of the same age or younger	156 (5.5)	185 (21.4)	362 (6.0)	262 (9.0)
Male partner is 1–4 years older	1242 (43.6)	283 (32.8)	2105 (34.9)	1069 (36.6)
Male partner is 5 years older	320 (11.2)	76 (8.7)	675 (11.2)	294 (10.1)
Male partner is 6–9 years older	753 (26.4)	176 (20.3)	1883 (31.2)	817 (28.0)
Male partner is 10 years older or more	378 (13.3)	145 (16.7)	1014 (16.8)	475 (16.3)
Educational difference with partner^c^				
Male partner has fewer years	1107 (38.9)	305 (35.2)	2338 (38.7)	932 (31.9)
Partners have the same years	462 (16.2)	223 (25.8)	1206 (20.0)	974 (33.4)
Male partner has 1–3 more years	901 (31.6)	281 (32.5)	1754 (29.0)	810 (27.8)
Male partner has 4 or more years	378 (13.3)	56 (6.5)	741 (12.3)	201 (6.9)
High empowerment overall	1398 (49.1)	388 (44.9)	3160 (52.3)	1023 (35.1)
Low empowerment overall	1451 (50.9)	477 (55.1)	2879 (47.7)	1894 (64.9)
Man has nonmarital sexual partners^d^				
Yes	178 (6.3)	74 (8.6)	826 (13.7)	323 (11.1)
No	2671 (93.8)	791 (91.4)	5213 (86.3)	2594 (88.9)
Woman can ask a man to wear a condom given an STI				
Yes	2444 (85.8)	836 (96.7)	5098 (84.4)	2414 (82.8)
No	405 (14.2)	29 (3.3)	941 (15.6)	503 (17.2)
Woman has the ability to refuse sex				
Yes	2094 (73.5)	804 (93.0)	4230 (70.1)	2187 (75.0)
No	755 (26.5)	61 (7.0)	1809 (29.9)	730 (25.0)
	**Mean (SD)**	**Mean (SD)**	**Mean (SD)**	**Mean (SD)**
Age difference with partner in years	5.3 (4.1)	4.5 (6.1)	5.9 (4.3)	5.6 (4.7)

+All data are weighted.

^a^ Polygamous union refers to whether the man has more than one wife.

^b^ The age difference is calculated as the respondent's age subtracted from the male partner's reported age.

^c^ The educational difference is calculated as the respondent's years of education subtracted from the male partner's years of education.

^d^ Multiple sexual partnerships refer to the man having sex with more than one woman, not including the wife/partner, in the past 12 months.

SD, standard deviation; STI, sexually transmitted infection.

### Multiple sexual partnerships

**FIG. 2.**
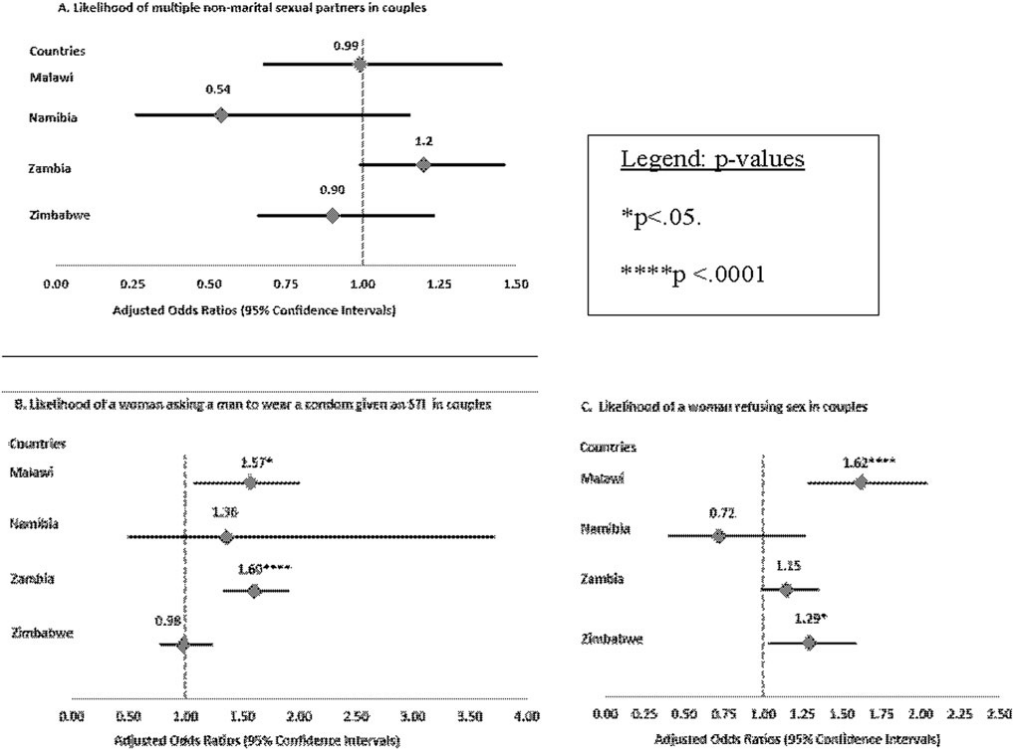
Multivariable logistic regression assessing the association between women's empowerment and outcomes in couples aged 15–64 years.

In Namibia, women who were involved in household decision-making (UOR = 0.48, *p* = 0.0182) and women who had the same or more education than their partners (UOR = 0.44, *p* = 0.0078) were less likely to experience their male partner having multiple sexual partners in the relationship (Table 2). In Zambia, women who were economically independent were less likely to have a spouse with multiple sexual partners compared with women who were economically dependent (UOR = 0.69, *p* = 0.0004) (Table 2). Based on the findings from the multivariable model (Fig. 2A), high levels of empowerment in women were not associated with the likelihood of the male partner having multiple sexual partners across all countries.

**Table 2.** Relationship of Female Empowerment Indicators to Likelihood of Male Nonmarital Multiple Sexual Partners in South African Couples Aged 15–64 Years^a^

	Malawi 2010 (*n* = 2849)	Namibia 2013 (*n* = 865)	Zambia 2013–14 (*n* = 6039)	Zimbabwe 2010–11 (*n* = 2917)
	Odds ratio (UOR) (95% CI), *p*	Odds ratio (UOR) (95% CI), *p*	Odds ratio (UOR) (95% CI), *p*	Odds ratio (UOR) (95% CI), *p*
SDL				
Decision-making^b^	0.63 (0.36–1.10), 0.1064	0.48 (0.26–0.88), 0.0182	0.93 (0.77–1.11), 0.4104	0.93 (0.69–1.25), 0.6161
Economic independence^c^	1.38 (0.97–1.96), 0.0743	1.22 (0.66–2.26), 0.5161	0.69 (0.56–0.85), 0.0004	1.09 (0.82–1.45), 0.5536
SDP				
Wife-beating attitudes^d^	0.98 (0.60–1.59), 0.9347	0.67 (0.36–1.23), 0.1928	1.19 (0.98–1.45), 0.0743	0.85 (0.64–1.13), 0.2617
Age difference^e^	1.10 (0.66–1.83), 0.7256	0.80 (0.37–1.69), 0.5502	1.93 (1.45–2.57), <0.0001	1.80 (1.17–2.78), 0.0081
Education difference^f^	0.96 (0.67–1.39), 0.8361	0.44 (0.24–0.80), 0.0078	1.13 (0.94–1.35), 0.1947	0.84 (0.63–1.12), 0.2332
Women's empowerment index SDL^g^ (high vs. low)	1.00 (0.70–1.41), 0.9757	0.86 (0.44–1.68), 0.6599	0.65 (0.52–0.81), 0.0001	1.05 (0.72–1.52), 0.8193
Women's empowerment index SDP^h^ (high vs. low)	0.85 (0.60–1.21), 0.3660	0.32 (0.14–0.70), 0.0043	1.51 (1.24–1.84), <0.0001	0.89 (0.65–1.22), 0.4601
Women's empowerment all indicators^i^ (high vs. low)	0.93 (0.65–1.32), 0.6633	0.49 (0.26–0.93), 0.0296	1.19 (0.98–1.44), 0.0824	0.83 (0.60–1.13), 0.2282

^a^ All data are weighted.

^b^ The woman is involved alone or jointly versus uninvolved in decisions.

^c^ The woman is currently working versus she did not work in the past 12 months.

^d^ The woman agrees with none of the scenarios versus she agrees with at least one wife-beating scenario.

^e^ The man is younger or up to 9 years older than the woman versus the man is 10 years older or more.

^f^ The man has fewer or the same years of education as the woman versus the man has more years of education.

^g^ This index is the SDL construct with decision-making and economic dependence.

^h^ This index is the SDP construct with wife-beating attitudes, educational differences, and age differences.

^i^ This index includes all TGP construct indicators combined.

CI, confidence interval; SDL, sexual division of labor; SDP, sexual division of power; TGP, Theory of Gender and Power.

### Self-efficacy for safer sex negotiation and sex refusal

Women with economic independence were more likely to negotiate safer sex with partners compared with those who were economically dependent in Malawi (UOR = 1.39, *p* = 0.0207), Namibia (UOR = 2.44, *p* = 0.0395), and Zimbabwe (UOR = 2.12, *p* < 0.0001) (Table 3). Moreover, Zambian women who had sole/joint involvement versus no involvement in household decisions and rejected versus condoned wife-beating were more likely (36% and 52%, respectively) to say they/a woman could negotiate safer sex (Table 3).

**Table 3.** Relationship of Female Empowerment Indicators to Likelihood of Safer Sex Negotiation Given a Sexually Transmitted Infection in South African Couples Aged 15–64 Years^a^

	Malawi 2010 (*n* = 2883)	Namibia 2013 (*n* = 865)	Zambia 2013–14 (*n* = 6039)	Zimbabwe 2010–11 (*n* = 2917)
	Odds ratio (UOR) (95% CI), *p*	Odds ratio (UOR) (95% CI), *p*	Odds ratio (UOR) (95% CI), *p*	Odds ratio (UOR) (95% CI), *p*
SDL				
Decision-making^b^	0.82 (0.53–1.26), 0.3632	1.32 (0.61–2.86), 0.4772	1.36 (1.14–1.62), 0.0005	0.94 (0.74–1.19), 0.5987
Economic independence^c^	1.39 (1.05–1.83), 0.0207	2.44 (1.04–5.68), 0.0395	1.15 (0.95–1.40), 0.1631	2.12 (1.60–2.80), <0.0001
SDP				
Wife-beating attitudes^d^	1.42 (0.95–2.11), 0.0905	1.57 (0.65–3.75), 0.3140	1.52 (1.28–1.81), <0.0001	1.10 (0.88–1.37), 0.4090
Age difference^e^	0.53 (0.35–0.80), 0.0028	2.06 (0.89–4.78), 0.0932	1.13 (0.89–1.44), 0.3068	1.06 (0.81–1.40), 0.6662
Education difference^f^	1.20 (0.92–1.56), 0.1857	1.42 (0.67–3.01), 0.3577	1.07 (0.90–1.28), 0.4270	0.97 (0.76–1.24), 0.8165
Women's empowerment index SDL^g^ (high vs. low)	1.36 (1.02–1.83), 0.0398	1.91 (0.67–5.46), 0.2245	1.34 (1.09–1.66), 0.0060	1.88 (1.37–2.58), 0.0001
Women's empowerment index SDP^h^ (high vs. low)	1.14 (0.87–1.50), 0.3479	1.17 (0.50–2.75), 0.7111	1.50 (1.26–1.78), <0.0001	1.09 (0.89–1.33), 0.4222
Women's empowerment all indicators^i^ (high vs. low)	1.53 (1.12–2.07), 0.0068	2.51 (0.98–6.43), 0.0542	1.74 (1.47–2.05), <0.0001	1.13 (0.90–1.41), 0.3041

^a^ All data are weighted.

^b^ The woman is involved alone or jointly versus uninvolved in decisions.

^c^ The woman is currently working versus she did not work in the past 12 months.

^d^ The woman agrees with none of the scenarios versus she agrees with at least one wife-beating scenario.

^e^ The man is younger or up to 9 years older than the woman versus the man is 10 years older or more.

^f^ The man has fewer or the same years of education as the woman versus the man has more years of education.

^g^ This index is the SDL construct with decision-making and economic dependence.

^h^ This index is the SDP construct with wife-beating attitudes, educational differences, and age differences.

^i^ This index includes all TGP construct indicators combined.

Adjusted models showed that high levels of empowerment in women were associated with higher odds of safer sex negotiation in Malawi (AOR = 1.57, *p* < 0.05) and Zambia (AOR = 1.60, *p* < 0.0001) (Fig. 2B). Finally, women who had sole/joint involvement in household decisions (Zimbabwe), had economic independence (Malawi and Zimbabwe), and rejected at least one reason for wife-beating (Namibia, Zambia, and Zimbabwe) were more likely to refuse sex (Table 4). Per multivariable models, high women's empowerment was associated with an increase in the likelihood of sex refusal in Malawi (AOR = 1.62, 95% *p* < 0.0001) and Zimbabwe (AOR = 1.29, *p* < 0.05) (Fig. 2C).

**Table 4.** Relationship of Female Empowerment Indicators to Likelihood of Sex Refusal in South African Couples Aged 15–64 Years^a^

	Malawi 2010 (*n* = 2883)	Namibia 2013 (*n* = 865)	Zambia 2013–14 (*n* = 6039)	Zimbabwe 2010–11 (*n* = 2917)
	Odds ratio (UOR) (95% CI), *p*	Odds ratio (UOR) (95% CI), *p*	Odds ratio (UOR) (95% CI), *p*	Odds ratio (UOR) (95% CI), *p*
SDL				
Decision-making^b^	1.32 (0.96–1.82), 0.0889	1.01 (0.58–1.76), 0.9866	1.01 (0.87–1.17), 0.9235	1.25 (1.03–1.53), 0.0278
Economic independence^c^	1.59 (1.29–1.96), <0.0001	1.35 (0.78–2.34), 0.2795	1.04 (0.92–1.22), 0.4170	1.56 (1.27–1.92), <0.0001
SDP				
Wife-beating attitudes^d^	1.23 (0.90–1.69), 0.2013	1.97 (1.11–3.51), 0.0216	1.20 (1.03–1.40), 0.0209	1.25 (1.01–1.55), 0.0402
Age difference^e^	1.16 (0.86–1.56), 0.3293	1.18 (0.60–2.31), 0.6400	1.15 (0.95–1.39), 0.1417	1.05 (0.81–1.35), 0.7368
Education difference^f^	1.11 (0.90–1.37), 0.3221	1.34 (0.77–2.33), 0.2986	1.04 (0.90–1.19), 0.6317	1.00 (0.81–1.22), 0.9621
Women's empowerment index SDL^g^ (high vs. low)	1.46 (1.16–1.85), 0.0016	1.00 (0.54–1.86), 1.000	1.08 (0.93–1.26), 0.3224	1.57 (1.25–1.99), 0.0002
Women's empowerment index SDP^h^ (high vs. low)	1.34 (1.08–1.66), 0.0086	1.15 (0.64–2.08), 0.6437	1.19 (1.02–1.38), 0.0253	1.20 (0.98–1.48), 0.0847
Women's empowerment all indicators^i^ (high vs. low)	1.64 (1.31–2.06), <0.0001	0.98 (0.54–1.77), 0.9401	1.10 (0.95–1.28), 0.2166	1.39 (1.13–1.71), 0.0021

^a^ All data are weighted.

^b^ The woman is involved alone or jointly versus uninvolved in decisions.

^c^ The woman is currently working versus she did not work in the past 12 months.

^d^ The woman agrees with none of the scenarios versus she agrees with at least one wife-beating scenario.

^e^ The man is younger or up to 9 years older than the woman versus the man is 10 years older or more.

^f^ The man has fewer or the same years of education as the woman versus the man has more years of education.

^g^ This index is the SDL construct with decision-making and economic dependence.

^h^ This index is the SDP construct with wife-beating , educational differences, and age differences.

^i^ This index includes all TGP construct indicators combined.

## Discussion

This investigation evaluated associations between high empowerment and HIV-relevant sexual risk behaviors in married/cohabitating women from four countries in SSA. This study represents a novel assessment of empowerment and HIV risk behaviors in couples using the TGP as a framework. Our hypothesis was confirmed by the association between high levels of women's empowerment and increased odds for indicators of safer sex negotiation and sex refusal, although this finding was not universal.

Women's empowerment in coupled relationships was associated with safer sex negotiation in Malawi and Zambia and sex refusal in Malawi and Zimbabwe. The observations of safer sex negotiation are in line with studies in the United States of America,^40,41^ Eastern and Southern Africa,^42–47^ and Nepal.^48^ Other DHS studies reflected similar findings regarding decision-making involvement and the increased likelihood of sex refusal in Nepal^48^ and Cambodia.^49^ Unlike these studies, our research used a multidimensional construct that illustrated which risk factors and exposures lead to sexual divisions and HIV risk behaviors. Finally, the high frequency of women's responses for the ability to initiate condom use and to refuse sex calls into question the predominant “female victim, male perpetrator” discourse.^11,12^

The key drivers of empowerment associated with an increased likelihood of safer sex negotiation and sex refusal were economic independence, sole or co-participation in household decision-making, and a negative attitude toward wife-beating for any reason. These results confirm the interconnectedness of gender power relations, control of resources, GBV, and women's HIV risk in African women.^50^ These findings also suggest that women with decision-making involvement, economic independence, and equitable gender-role attitudes have agency and resources^23^ that, in turn, reduce burdens from power imbalances and influence safer sexual practices in relationships.^17^

Our results differed from research in the United States, Cambodia, and South Africa. Researchers found no association between relationship control and condom use initiation in Asian American women.^51^ However, that study measured relationship control with the Sexual Relationship Power Scale, which does not include all constructs of the TGP. In Cambodian and South African couples, increases in egalitarian norms decreased the likelihood of condom use due to trust and lowered perception of HIV risk.^49,52^ In contrast to those studies, we incorporated a question about asking a partner to wear a condom given an STI to emphasize self-efficacy in the context of disease risk and prevention.

The fact that our results for associations between women's empowerment and self-efficacy outcomes varied across all countries is also noteworthy. Researchers hypothesize that women in SSA who are involved in household decisions, reject intimate partner violence, and support sexual rights may still have less control over their sexual and reproductive health in a relationship.^23^ In addition, reviews and studies around the world assert that condom use involves a complex web of dynamics among men and women.^45,53–55^ In countries with generalized HIV epidemics, other interpersonal power gradients and cultural norms not captured in this analysis may affect safer sex choices.^56,57^

Contrary to our hypothesis, we found no significant associations between high levels of empowerment status in women and a decrease in the likelihood of multiple sexual partnerships by men. The overall finding is consistent with studies on marital subordination, interpersonal power, female monogamy, male multiple sexual partners, and HIV risk across SSA.^28,57–59^ This study finding also suggests an acceptance of social and cultural norms for masculinity, namely, “acquiesced femininity” (*e.g.*, acceptance of men's dominance, control of economic resources, and multiple partners), regardless of a woman's empowerment status.^12,60–63^

Our findings differed from those of a multicountry DHS study in Gabon, Mozambique, Sierra Leone, and Zambia, a study in Cameroon, and DHS research in Eastern Africa reporting associations between women's empowerment and an increased likelihood of multiple sexual partnerships and HIV risk.^56,64,65^ Of note, those studies included women regardless of marital status, chose countries with varied HIV prevalence, omitted men's sexual behaviors, and confined empowerment indicators to educational or economic dimensions. In addition, researchers in prior investigations have argued that empowerment indicators such as decision-making involvement may not reflect actual empowerment if women still carry the brunt of home responsibilities.^23,66,67^

This study has many strengths to consider. First, the large sample size in each country provided enough power to provide more precise estimates in multivariable models. Second, the application of weights in the analysis made the results generalizable to similar couples in each country. Third, countries with high HIV prevalence provided context for existing and future HIV prevention initiatives. Fourth, the consistent pattern of indicators that influenced empowerment by country is noteworthy for future couple-level interventions for HIV prevention. Finally, this is the first known study to apply TGP concepts to assess empowerment and HIV risk behaviors using couples as the unit of analysis in an African context.

We must consider some limitations in this study that should lead to a cautious interpretation of our results. The cross-sectional nature of this analysis limits causal inference, so we are unable to determine whether high empowerment in women led to sexual behaviors or vice versa. Next, social desirability and recall biases could occur, as respondents may underreport pre- or extramarital relationships and may not remember details that occurred in the past year.

Although we evaluated polygamy and place of residence as proxies for the impact of traditional norms in the community, other contextual variables could influence associations. In addition, all countries had missing data or lacked variability in responses to empowerment indicators and outcomes, which could have influenced statistical power for finding significant associations in multivariable models. In the future, we recommend couple-level HIV prevention research with longitudinal analyses of data that are nationally representative.

Overall, this study adds to the body of knowledge on the role of gender-based power inequity within heterosexual relationships as determinants of HIV-relevant risk behaviors and transmission among couples in SSA. This understanding of modifiable gender dynamics in SSA couples is vital for reducing the high burden of HIV acquisition and HIV-related disability for women aged 15–49 years in the region.^68^ Furthermore, gaining a nuanced understanding of empowerment indicators improves health messaging in HIV prevention programs aimed at repurposing social and cultural norms in association with risky sexual behaviors.^69^

Policymakers should consider empowerment indicators and prioritize women and girls in national strategic plans relevant to their country and context. National governments should continue to promote economic empowerment, rural development, education, health, and equality policies for men and women while enforcing laws against GBV. Our results have other implications as well. The study results provide an opportunity for national governments and policymakers to use frameworks such as the TGP to target social, economic, and health policy in ways that minimize lack of autonomy, GBV, and poverty as structural drivers of high-risk behaviors in men and women. Thus, these initiatives can address in turn HIV-related risk behaviors and risks among women and girls in multiple sub-Saharan African countries.

We also recommend that governments boost spending with investments from the private sector and nongovernmental organizations for behavioral interventions to understand power dynamics in couples and facilitate empowerment. In the future, these results can influence existing and future couple-level interventions for HIV prevention, such as serodiscordant studies and couples-based HIV testing and counseling programs. Finally, these results provide important context to evaluate results from ongoing interventions such as Stepping Stones-Creating Futures in South Africa,^70^ the DREAMS Initiative in young adults and adolescents,^71^ and the Malawi BRIDGE Project.^72^

## Conclusions

In summary, our research assessed the association between TGP constructs for empowerment in married/cohabitating women and HIV risk behaviors. Among women in heterosexual relationships, high empowerment was associated with higher odds of safer sex negotiation in Malawi and Zambia and with sex refusal in Malawi and Zimbabwe. Indicators of household decision-making involvement, female economic independence, and rejecting all reasons for wife-beating contributed strongly to these associations. These findings provide evidence that, per the TGP, constructs of sexual divisions among couples influence HIV risk in Eastern and Southern Africa. Policy and development officials in SSA should consider these key indicators as targets for future interventions to promote gender equality and address HIV risk among couples.

## Ethical Approval and Consent to Participate

National ethics boards review DHS surveys, and ICF International's institutional review board approves data collection procedures. All respondents gave informed consent for surveys and HIV testing. The study was exempt from full institutional board review by the University of Georgia because of the use of anonymized secondary data.

## Abbreviations Used

AOR: adjusted odds ratio
CI: confidence interval
DHS: Demographic and Health Survey
GBV: gender-based violence
HIV: human immunodeficiency virus
SD: standard deviation
SDL: sexual division of labor
SDP: sexual division of power
STI: sexually transmitted infection
SSA: sub-Saharan Africa
TGP: Theory of Gender and Power
UOR: unadjusted odds ratio

## References

[1] Joint United Nations Programme on HIV/AIDS. UNAIDS global HIV statistics fact sheet 2019. Available at: https://www.unaids.org/sites/default/files/media_asset/UNAIDS_FactSheet_en.pdf Published November 2019 Accessed 1130, 2019

[2] Joint United Nations Programme on HIV/AIDS. UNAIDS GAP report 2014, 2014 Available at: http://files.unaids.org/en/media/unaids/contentassets/documents/unaidspublication/2014/UNAIDS_Gap_report_en.pdf Accessed 31, 2018

[3] United Nations Joint Programme on HIV/AIDS. UNAIDS data book 2017, 2017 Available at: https://www.unaids.org/en/resources/documents/2017/2017_data_book Accessed 35, 2018

[4] Richardson ET, Collins SE, Kung T, et al. Gender inequality and HIV transmission: A global analysis. J Int AIDS Soc 2014;17:19035 24976436 10.7448/IAS.17.1.19035 PMC4074603

[5] Glick PJ, Sahn DE Are Africans practicing safer sex? Evidence from demographic and health surveys for eight countries. Econ Dev Cult Change 2008;56:397–439

[6] Onsomu EO, Kimani JK, Abuya BA, et al. Delaying sexual debut as a strategy for reducing HIV epidemic in Kenya. Afr J Reprod Health 2013;17:46–57 24069751

[7] Chimoyi LA, Musenge E Spatial analysis of factors associated with HIV infection among young people in Uganda, 2011. BMC Public Health 2014;14:555 24898872 10.1186/1471-2458-14-555 PMC4061924

[8] Wekwete NN, Sanhokwe H, Murenjekwa W, Takavarasha F, Madzingira N. Spousal gender-based violence and women's empowerment in the 2010-11 Zimbabwe Demographic and Health Survey, 2014 Available at: http://dhsprogram/com/pubs/pdf/WP108/WP108.pdf DHS Working Papers no. 108 (Zimbabwe Working Papers no. 9) Accessed 39, 2018

[9] Hunter DJ. AIDS in sub-Saharan Africa: The epidemiology of heterosexual transmission and the prospects for prevention. Epidemiology 1993;4:63–72 8420583

[10] Kishor S, Subaiya L. Understanding women's empowerment: A comparative analysis of demographic and health surveys (DHS) data, 2008 Available at: https://dhsprogram.com/pubs/pdf/CR20/CR20.pdf DHS Comparative Reports no. 20 Accessed 31, 2018

[11] Higgins JA, Hoffman S, Dworkin SL Rethinking gender, heterosexual men, and women's vulnerability to HIV/AIDS. Am J Public Health 2010;100:435–445 20075321 10.2105/AJPH.2009.159723 PMC2820057

[12] Wathuta J. Gender inequality dynamics in the prevention of a heterosexual HIV epidemic in sub-Saharan Africa. Afr J AIDS Res 2016;15:55–66 27002358 10.2989/16085906.2016.1150310

[13] Gupta GR, Parkhurst JO, Ogden JA, Aggleton P, Mahal A Structural approaches to HIV prevention. Lancet 2008;372:764–775 18687460 10.1016/S0140-6736(08)60887-9

[14] Jiwatram-Negron T, El-Bassel N Systematic review of couple-based HIV intervention and prevention studies: Advantages, gaps, and future directions. AIDS Behav 2014;18:1864 24980246 10.1007/s10461-014-0827-7 PMC4507500

[15] Connell RW Gender and power: Society, the person and sexual politics. Stanford, CA: Stanford University Press, 1987

[16] Ewerling F, Lynch JW, Victora CG, van Eerdewijk A, Tyszler M, Barros AJD The SWPER index for women's empowerment in Africa: Development and validation of an index based on survey data. Lancet Glob Health 2017;5:e916–e923 28755895 10.1016/S2214-109X(17)30292-9 PMC5554795

[17] Wingood GM, DiClemente RJ Application of the theory of gender and power to examine HIV-related exposures, risk factors, and effective interventions for women. Health Educ Behav 2000;27:539–565 11009126 10.1177/109019810002700502

[18] Gibbs A, Crone ET, Willan S, Mannell J The inclusion of women, girls and gender equality in National Strategic Plans for HIV and AIDS in southern and eastern Africa. Glob Public Health 2012;7:1120–1144 22812919 10.1080/17441692.2012.701319

[19] Fisher A, Way A The demographic and health surveys program: An overview. Int Fam Plan Perspect 1988;14:15–19

[20] Mishra V, Vaessen M, Boerma JT, et al. HIV testing in national population-based surveys: Experience from the Demographic and Health Surveys. Bull World Health Organ 2006;84:537 16878227 10.2471/blt.05.029520 PMC2627390

[21] Demographic and Health Surveys [Internet]. ICF International, 2012 Available at: https://dhsprogram.com/ Accessed 1119, 2015

[22] Suzuki S. CPH study session—Biostatistics. University of North Texas Health Science Center School of Public Health, 2017 Available at: https://s3.amazonaws.com/nbphe-wp-production/app/uploads/2017/05/Biostatistics_2017-2.pdf Accessed 717, 2017

[23] Upadhyay UD, Karasek D. Women's empowerment and achievement of desired fertility in Sub-Saharan Africa, 2010 Available at: https://pdfs.semanticscholar.org/0f9a/08ff7246eaf63d230a409e36be15018f7505.pdf DHS Working Papers no. 80 Accessed 12, 2018

[24] Luke N. Age and economic asymmetries in the sexual relationships of adolescent girls in sub-Saharan Africa. Stud Fam Plann 2003;34:67–86 12889340 10.1111/j.1728-4465.2003.00067.x

[25] Leclerc-Madlala S. Age-disparate and intergenerational sex in southern Africa: The dynamics of hypervulnerability. AIDS 2008;22(Suppl. 4):S17–S25 10.1097/01.aids.0000341774.86500.53 19033752

[26] Maughan-Brown B, Evans M, George G Sexual behaviour of men and women within age-disparate partnerships in South Africa: Implications for young women's HIV risk. PLoS One 2016;11:e0159162 27526116 10.1371/journal.pone.0159162 PMC4985138

[27] Zuma K, Shisana O, Rehle TM, et al. New insights into HIV epidemic in South Africa: Key findings from the National HIV Prevalence, Incidence and Behaviour Survey, 2012. Afr J AIDS Res 2016;15:67–75 27002359 10.2989/16085906.2016.1153491

[28] Chiao C, Mishra V, Ksobiech K Spousal communication about HIV prevention in Kenya. J Health Commun 2011;16:1088–1105 21644167 10.1080/10810730.2011.571335

[29] Wojcicki JM. Socioeconomic status as a risk factor for HIV infection in women in East, Central and Southern Africa: A systematic review. J Biosoc Sci 2005;37:1–36 15688569 10.1017/s0021932004006534

[30] Harling G, Barnighausen T The role of partners' educational attainment in the association between HIV and education amongst women in seven sub-Saharan African countries. J Int AIDS Soc 2016;19:20038 26902392 10.7448/IAS.19.1.20038 PMC4762222

[31] Magadi MA. Understanding the gender disparity in HIV infection across countries in sub-Saharan Africa: Evidence from the Demographic and Health Surveys. Sociol Health Illn 2011;33:522–539 21545443 10.1111/j.1467-9566.2010.01304.x PMC3412216

[32] Hargreaves JR, Glynn JR Educational attainment and HIV-1 infection in developing countries: A systematic review. Trop Med Int Health 2002;7:489–498 12031070 10.1046/j.1365-3156.2002.00889.x

[33] Fox AM. The HIV-poverty thesis re-examined: Poverty, wealth or inequality as a social determinant of HIV infection in sub-Saharan Africa? J Biosoc Sci 2012;44:459–480 22273351 10.1017/S0021932011000745

[34] Chen L, Jha P, Stirling B, et al. Sexual risk factors for HIV infection in early and advanced HIV epidemics in sub-Saharan Africa: Systematic overview of 68 epidemiological studies. PLoS One 2007;2:e1001 17912340 10.1371/journal.pone.0001001 PMC1994584

[35] Vorster HH, Wissing MP, Venter CS, et al. The impact of urbanization on physical, physiological and mental health of Africans in the North West Province of South Africa: The THUSA study. S Afr J Sci 2000;96:505–514

[36] Connolly C, Shisana O, Colvin M, Stoker D Epidemiology of HIV in South Africa—Results of a national, community-based survey. S Afr Med J 2004;94:776–781 15487845

[37] Reniers G, Tfaily R Polygyny, partnership concurrency, and HIV transmission in Sub-Saharan Africa. Demography 2012;49:1075–1101 22661302 10.1007/s13524-012-0114-z

[38] Fox AM. Marital concurrency and HIV risk in 16 African countries. AIDS Behav 2014;18:791–800 24398590 10.1007/s10461-013-0684-9

[39] SAS 9.4(c) [software]. Statistical Analysis System (SAS) Institute, Inc., 2013 July [downloaded 2016 Aug 5]

[40] Pulerwitz J, Amaro H, De Jong W, Gortmaker SL, Rudd R Relationship power, condom use and HIV risk among women in the USA. AIDS Care 2002;14:789–800 12511212 10.1080/0954012021000031868

[41] VanderDrift LE, Agnew CR, Harvey SM, Warren JT Whose intentions predict? Power over condom use within heterosexual dyads. Health Psychol 2013;32:1038–1046 23025301 10.1037/a0030021

[42] Greig FE, Koopman C Multilevel analysis of women's empowerment and HIV prevention: Quantitative survey results from a preliminary study in Botswana. AIDS Behav 2003;7:195–208 14586204 10.1023/a:1023954526639

[43] Pettifor AE, Measham DM, Rees HV, Padian NS Sexual power and HIV risk, South Africa. Emerg Infect Dis 2004;10:1996–2004 15550214 10.3201/eid1011.040252 PMC3328992

[44] Langen TT. Gender power imbalance on women's capacity to negotiate self-protection against HIV/AIDS in Botswana and South Africa. Afr Health Sci 2005;5:188–197 16245988 10.5555/afhs.2005.5.3.188 PMC1831928

[45] Boer H, Mashamba MT Gender power imbalance and differential psychosocial correlates of intended condom use among male and female adolescents from Venda, South Africa. Br J Health Psychol 2007;12:51–63 17288665 10.1348/135910706X102104

[46] Exavery A, Kante AM, Jackson E, et al. Role of condom negotiation on condom use among women of reproductive age in three districts in Tanzania. BMC Public Health 2012;12:1097 23256530 10.1186/1471-2458-12-1097 PMC3585459

[47] De Coninck Z, Feyissa IA, Ekstrom AM, Marrone G Improved HIV awareness and perceived empowerment to negotiate safe sex among married women in Ethiopia between 2005 and 2011. PLoS One 2014;9:e115453 25506823 10.1371/journal.pone.0115453 PMC4266675

[48] Atteraya MS, Kimm H, Song IH Women's autonomy in negotiating safer sex to prevent HIV: Findings from the 2011 Nepal Demographic and Health Survey. AIDS Educ Prev 2014;26:1–12 24450274 10.1521/aeap.2014.26.1.1

[49] Ung M, Boateng GO, Armah FA, Amoyaw JA, Luginaah I, Kuuire V Negotiation for safer sex among married women in Cambodia: The role of women's autonomy. J Biosoc Sci 2014;46:90–106 23517629 10.1017/S0021932013000151

[50] Kathewera-Banda M, Gomile-Chidyaonga F, Hendriks S, Kachika T, Mitole Z, White S Sexual violence and women's vulnerability to HIV transmission in Malawi: A rights issue. Int Soc Sci J 2005;57:649–660

[51] Hahm HC, Lee J, Rough K, Strathdee SA Gender power control, sexual experiences, safer sex practices, and potential HIV risk behaviors among young Asian-American women. AIDS Behav 2012;16:179–188 21259042 10.1007/s10461-011-9885-2 PMC3389795

[52] Harrison A, O'Sullivan LF, Hoffman S, Dolezal C, Morrell R Gender role and relationship norms among young adults in South Africa: Measuring the context of masculinity and HIV risk. J Urban Health 2006;83:709–722 16758334 10.1007/s11524-006-9077-y PMC2430491

[53] Blanc AK. The effect of power in sexual relationships on sexual and reproductive health: An examination of the evidence. Stud Fam Plann 2001;32:189–213 11677692 10.1111/j.1728-4465.2001.00189.x

[54] Woolf SE, Maisto SA Gender differences in condom use behavior? The role of power and partner-type. Sex Roles 2008;58:689–701

[55] Maticka-Tyndale E. Condoms in sub-Saharan Africa. Sex Health 2012;9:59–72 22348634 10.1071/SH11033

[56] Abimanyi-Ochom J. The better the worse: Risk factors for HIV infection among women in Kenya and Uganda: Demographic and health survey. AIDS Care 2011;23:1545–1550 22117124 10.1080/09540121.2011.582477

[57] Andersson N, Cockcroft A Choice-disability and HIV infection: A cross sectional study of HIV status in Botswana, Namibia and Swaziland. AIDS Behav 2012;16:189–198 21390539 10.1007/s10461-011-9912-3 PMC3254870

[58] Hageman KM, Dube HM, Mugurungi O, Gavin LE, Hader SL, St Louis ME Beyond monogamy: Opportunities to further reduce risk for HIV infection among married Zimbabwean women with only one lifetime partner. AIDS Behav 2010;14:113–124 19685181 10.1007/s10461-009-9603-5

[59] Buve A, Bishikwabo-Nsarhaza K, Mutangadura G The spread and effect of HIV-1 infection in sub-Saharan Africa. Lancet 2002;359:2011–2017 12076570 10.1016/S0140-6736(02)08823-2

[60] Brown J, Sorrell J, Raffaelli M An exploratory study of constructions of masculinity, sexuality and HIV/AIDS in Namibia, Southern Africa. Cult Health Sex 2005;7:585–598 16864224 10.1080/13691050500250198

[61] Simpson A. Sons and fathers/boys to men in the time of AIDS: Learning masculinity in Zambia. J South Afr Stud 2005;31:569–586

[62] Bingenheimer JB. Men's multiple sexual partnerships in 15 Sub-Saharan African countries: Sociodemographic patterns and implications. Stud Fam Plann 2010;41:1–17 21151707 10.1111/j.1728-4465.2010.00220.x PMC2998893

[63] Jewkes R, Morrell R Gender and sexuality: Emerging perspectives from the heterosexual epidemic in South Africa and implications for HIV risk and prevention. J Int AIDS Soc 2010;13:6 20181124 10.1186/1758-2652-13-6 PMC2828994

[64] Odimegwu CO, De Wet N, Banda PC Risky sexual behaviour among women: Does economic empowerment matter? Case of Gabon, Mozambique, Sierra-Leone and Zambia. Afr J AIDS Res 2016;15:333–340 27974018 10.2989/16085906.2016.1238401

[65] Mumah JN, Jackson-Smith D Do the factors associated with female HIV infection vary by socioeconomic status in Cameroon? J Biosoc Sci 2014;46:431–448 24871428 10.1017/S0021932013000631

[66] Mullany BC, Hindin MJ, Becker S Can women's autonomy impede male involvement in pregnancy health in Katmandu, Nepal? Soc Sci Med 2005;61:1993–2006 15922498 10.1016/j.socscimed.2005.04.006

[67] Hindin MJ. Women's input into household decisions and their nutritional status in three resource-constrained settings. Public Health Nutr 2006;9:485–493 16870021 10.1079/phn2005865

[68] Vos T, Allen C, Arora M, et al. Global, regional, and national incidence, prevalence, and years lived with disability for 310 diseases and injuries, 2015: A systematic analysis for the Global Burden of Disease Study 2015. Lancet 2016;388:1545–1602 27733282 10.1016/S0140-6736(16)31678-6 PMC5055577

[69] Banda F, Kunkeyani TE Renegotiating cultural practices as a result of HIV in the eastern region of Malawi. Cult Health Sex 2015;17:34–47 25138154 10.1080/13691058.2014.944569

[70] Gibbs A, Washington L, Willan S, et al. The stepping stones and creating futures intervention to prevent intimate partner violence and HIV-risk behaviours in Durban, South Africa: Study protocol for a cluster randomized control trial, and baseline characteristics. BMC Public Health 2017;17:336 28427380 10.1186/s12889-017-4223-x PMC5397780

[71] Office of the United States Global AIDS Coordinator. DREAMS innovation challenge fact sheet, 2014. Available at: www.pepfar.gov/documents/organization/247602.pdf Accessed 1127, 2017

[72] Kaufman MR, Rimal RN, Carrasco M, et al. Using social and behavior change communication to increase HIV testing and condom use: The Malawi BRIDGE Project. AIDS Care 2014;26(Suppl. 1):S46–S49 24735337 10.1080/09540121.2014.906741 PMC4427890

